# The interplay between sex, lifestyle factors and built environment on 20-year cardiovascular disease incidence; the ATTICA study (2002–2022)

**DOI:** 10.3389/fcvm.2024.1467564

**Published:** 2025-01-09

**Authors:** Evangelia G. Sigala, Christina Chrysohoou, Fotios Barkas, Evangelos Liberopoulos, Petros P. Sfikakis, Antigoni Faka, Costas Tsioufis, Christos Pitsavos, Demosthenes Panagiotakos

**Affiliations:** ^1^ Department of Nutrition and Dietetics, School of Health Sciences and Education, Harokopio University, Athens, Greece; ^2^ First Cardiology Clinic, Medical School, Hippokration Hospital, National and Kapodistrian University of Athens, Athens, Greece; ^3^ Department of Internal Medicine, Medical School, University of Ioannina, Ioannina, Greece; ^4^ First Department of Propaedeutic Internal Medicine, Medical School, Laiko General Hospital, National and Kapodistrian University of Athens, Athens, Greece; ^5^ Department of Geography, School of Environmental Sciences and Economics, Harokopio University, Athens, Greece

**Keywords:** cardiovascular diseases, epidemiology, risk assessment, built environment, urban-rural disparities, city planning, sex-specific differences

## Abstract

**Background and aim:**

This study aims to investigate the role of the built environment in terms of urban-rural disparities in cardiovascular disease (CVD) epidemiology, focusing on middle- and long-term CVD risk assessment. Moreover, this study seeks to explore sex-specific differences in urban and rural settings.

**Methods:**

The ATTICA Study is a prospective study conducted from 2002 onwards. At baseline, a random sample of 3,042 CVD-free adults (49.8% men) were randomly drawn from the population of the Attica region, in Greece, with 78% dwelling in urban and 22% in rural municipalities. Follow-up examinations were performed in 2006, 2012, and 2022. Of the total participants, 1,988 had complete data for CVD assessment in the 20-year follow-up.

**Results:**

The 10-year and 20-year CVD incidence was 11.8%, 28.0% in rural municipalities and 16.8%, 38.7% in urban municipalities, respectively (*p*s < 0.05). Unadjusted data analyses revealed significant differences in clinical, laboratory, and lifestyle-related CVD risk factors between urban and rural residents (*p*s < 0.05). Additionally, sex-based discrepancies in clinical, anthropometric, circulating, and lifestyle risk factors were observed in stratified analyses of urban and rural settings. Multivariate analyses, including generalized structural equation modeling (GSEM), revealed that the impact of the urban built environment on the long-term (20-year) CVD risk is mediated by lifestyle-related risk factors.

**Conclusion:**

Urban inhabitants exhibit a higher long-term CVD incidence compared to their rural counterparts, which was partially explained by their lifestyle behaviors. Targeted strategic city planning efforts promoting healthier lifestyle-related behaviors at the micro-environment level could potentially mitigate built-environment impacts on CVD health.

## 1 Introduction

Despite the progress in understanding the aetiopathology and management of cardiovascular disease (CVD), recent evidence from the Global Burden of Disease (GBD) 2021 report indicates that CVD, is the leading cause of disability-adjusted life years (DALYs), worldwide (1). In 2021, CVD was responsible for the loss of 428 million years of life lived in full health, with 244 million DALYs in males and 184 million DALYs in females, underscoring its immense burden. Moreover, CVD continues to be the primary driver of years of life lost (YLL) due to premature mortality and remains the principal cause of years of life lived with disability (YLD). Notably, while males experienced a downward trend in age-standardized incidence rates from 2010 to 2021, females demonstrated an upsurge, disputing the prevailing belief that CVD primarily affects males and highlighting the critical reality that females are “*understudied, under-recognized, underdiagnosed, and undertreated*” in the field of CVD medical research and clinical practice (2). Therefore, scientific communities endorse prioritizing sex- or gender-centric research, including data collection (2–7), to identify the underlying pathophysiological pathways and other less-known CVD predisposing factors, such as features of the built environment (2), acknowledging that sex (biological and physiological characteristics) and gender (socially constructed attributes) intersect with socio-economic inequalities, which in turn affect well-being and health outcomes (8), including CVD (2–6), thereby strengthening a vicious cycle that contributes to the disease burden (2).

The built environment, a subset of the human exposome, comprises all man-made or altered attributes of an individual's surroundings, such as green open spaces, buildings, and other infrastructure, and often accounts for the effects of the natural environment, including ambient air pollution, temperature, and noise pollution, all of which affect health-related outcomes during the lifespan (9). There is suggestive evidence that features of the micro-scale neighborhood-built environment in urban areas may constitute modifiable CVD predisposition factors (9, 10). Urban planning in newer cities, often designed around a car-centric model, results in poor infrastructure for public and pedestrian transportation, limited greenspaces, and increased exposure to environmental stressors (11). All these factors tend to promote a sedentary lifestyle, consequently leading to a higher CVD burden. In contrast, greenness and walkability in an urban neighborhood-built environment seem to offer a cardio-protective effect against CVD risk (10).

Urbanization, as a key indicator of urban growth (9), involves the rural-to-urban population migration and the transformation of prior rural areas to urban agglomerations, thereby inducing changes in lifestyle, culture, and primary income sources (12, 13). According to projections, approximately 7 in 10 people on Earth will live in an urban region by 2050, contributing to a population of 6.7 billion urban inhabitants (12). Conversely, rural population growth has substantially decreased, reaching its peak in 2021. This rapid urbanization, coupled with other factors, such as rising incomes and a demographic transition characterized by increases in life expectancy and declines in fertility rates, is one of the main drivers of nutritional transition, which entails shifting from traditional dietary patterns that are abundant in complex carbohydrate-based staple foods to energy-dense Western-type diets, leading to the obesity paradox and an increase in diet-related non-communicable diseases, including CVD (13, 14). Among others, urbanization impacts the structure of local markets by increasing the number and size of local food markets, either formal or informal retail, thus affecting the accessibility and availability of healthy food (13). Under the nexus of urbanization and food environments, two contradicting food environments may be defined. First, “*food swamps*” entail regions with substantial growth of informal retailers offering highly affordable, energy-dense, and low-quality foods; “*food deserts*” involve areas with limited availability of diverse, fresh, and nutritious foods, primarily due to restricted proximity to food businesses, potentially leading to caloric deficiency or energy surplus. Between these extremes of the spectrum of this nexus, urban areas exhibit varied patterns, ranging from high-density fast-food facilities offering ready, caloric-dense, and highly processed foods to areas with high-density food retailers that offer diverse, healthful foods (15). Besides lifestyle shifting, urbanization-induced natural environmental degradation has created a micro-scale climate in urban agglomerations, which is mainly characterized by phenomena, such as urban wind environment, urban heat islands, pollution dispersion due to thermal buoyancy, and high energy consumption from urban infrastructure (16). The aforementioned issues highlight an urgent need for primordial and primary CVD prevention within the framework of sustainable development, equity, diversity, and inclusion, particularly in urban settings, as people living in urban agglomerations often face greater exposure to environmental and psychosocial adversities (17).

**Figure 1:**
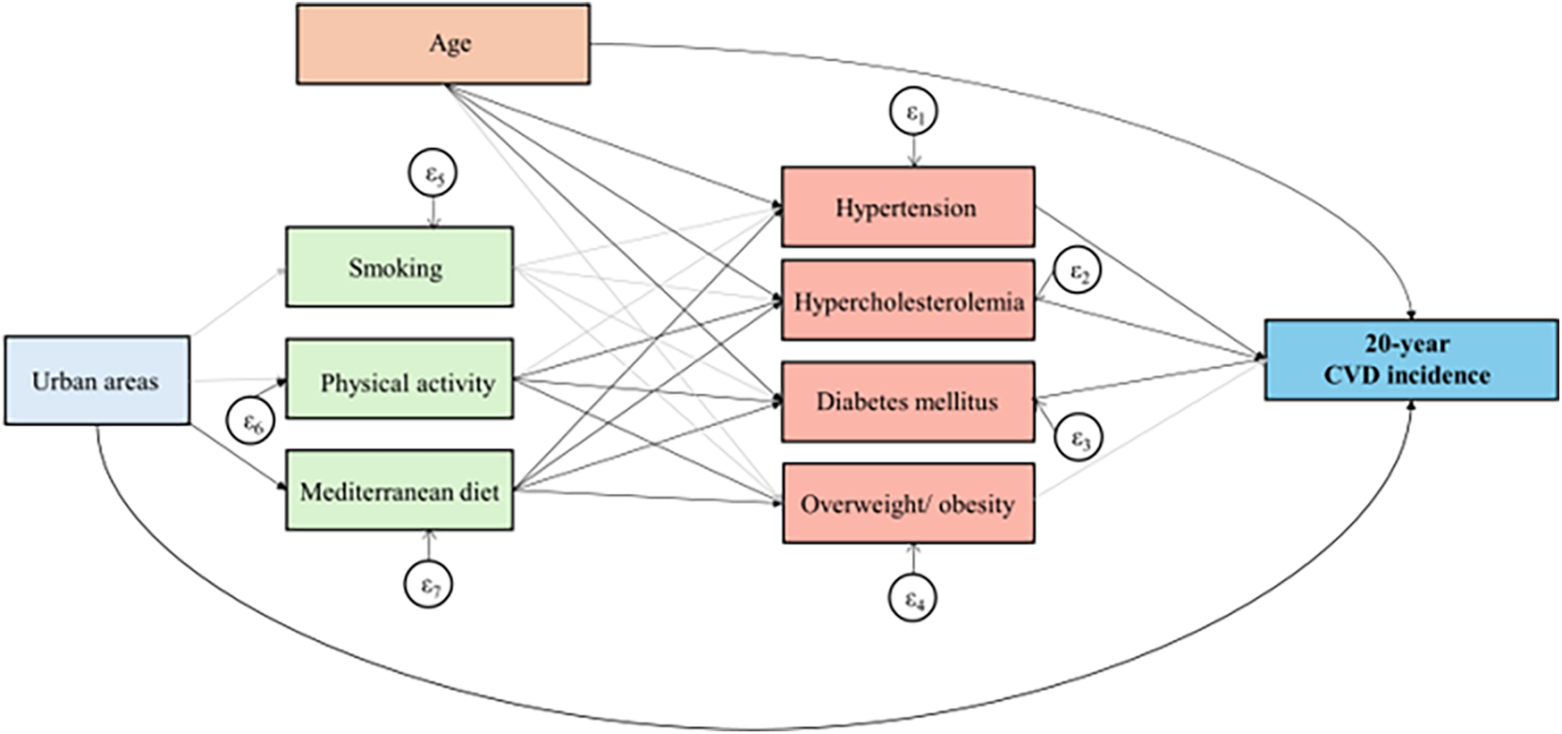
Results of the generalized structural equation models, that evaluated built environment in relation to 20-year CVD incidence in the ATTICA Study participants. Bold lines indicate significant paths and grey lines represent non-significant relationships.

The ATTICA Study (2002–2022) is one of the first long-term prospective studies that has utilized advanced spatial statistics techniques to map long-term CVD outcomes and report between-sex differences [*in publication* and (18)]. The present study aimed to explore the intricate pathways linking the living environment, lifestyle habits, and the 20-year CVD incidence, in light of a sex-based analysis. To achieve this, a generalized structural equation modeling approach was employed (Figure 1).

## 2 Materials and methods

### 2.1 Ethical approval, informed consent, and data confidentiality

The ATTICA Study conforms to the ethical principles stipulated by the Declaration of Helsinki and has obtained approval from the Ethics Committee of the First Cardiology Department of the National and Kapodistrian University of Athens (#017/01.05.2001) and the Ethics Committee of Harokopio University (#38/29.03.2022). Moreover, providing written informed consent from all participants was a prerequisite for their enrollment in the study, following a detailed presentation of the study's goals and procedures and guaranteeing the confidentiality of their data.

### 2.2 Study design and sample at baseline and at each follow-up examination

Initiated in 2001–2002 (19), the ATTICA Study is an ongoing epidemiological study that has already conducted three subsequent examinations: an intermediate 5-year follow-up in 2006 (20), a 10-year follow-up in 2012 (21), and a long-term 20-year follow-up in 2022 (22). The study's objectives included evaluating the presence of clinical and biochemical CVD predisposing factors in a representative sample of community-dwelling Greek individuals; investigating their correlations with socio-economic characteristics, lifestyle habits, and psychological traits of the sample; and assessing the prognostic value of these variables on CVD risk prediction (19, 21). To achieve these aims, a multi-stage, stratified random sampling was implemented, with strata established based on the population distribution of the Attica region, considering age (five age brackets), biological sex (males females), and Attica's subregions (27 municipalities), as determined by the 2001 Population and Housing Census. A representative cohort of 3,042 apparently healthy adults –without a previous diagnosis of CVD, cancer, or other inflammation-driven conditions– was recruited, with a participation rate of 75%. This baseline sample comprised 49.8% males and 50.2% females, with mean ages of 46 ± 13 and 45 ± 14 years old, respectively. In 2022, 2,169 individuals agreed to participate (participation rate at 71%), with 1,988 having complete data on CVD-related endpoints for the 20-year period.

At each examination (baseline and follow-up), participants were assessed in person either at their residences or their workplaces by trained health practitioners, following the standardized protocols of the ATTICA Study, as detailed in previous publications (19–22). Specifically, the study's subjects were interviewed regarding their socio-demographic characteristics, smoking habits, dietary intake, physical activity levels, engagement in leisure-time activities, anxiety and depression symptoms, and perceived health status, and they were also requested to report their personal and family medical history, along with any medication they were receiving. Moreover, at baseline, in addition to the questionnaires, interviewees underwent a series of physical examinations, including anthropometric and arterial blood pressure measurements. Additionally, morning venous blood samples were drawn from the antecubital veins after a 12 h period of fasting, during which participants refrained from food and drinks, including alcoholic beverages. For the follow-up evaluations, participants were initially contacted by phone to schedule a follow-up assessment (20–22). In cases where subjects had deceased before the subsequent examination, information was acquired from their family members and death registration certificates.

### 2.3 Assessment of fatal or non-fatal CVD events as primary endpoints

Outcome definitions were standardized based on the criteria of the 10th version of the WHO-International Classification of Diseases (ICD), while the WHO-ICD-9 coding was also retained to maintain consistency, as this version had been employed during the 5-year follow-up examination (19–22). Particularly, the primary endpoints encompassed non-fatal CVD episodes, including myocardial infarction or its complications within 28 days after the episode, angina pectoris, other ischemic heart disease, coronary revascularization procedures (such as coronary artery bypass or percutaneous coronary intervention), different types of heart failure, chronic arrythmias, ischemic stroke, and transient ischemic attack, as well as deaths attributable to CVD. Deaths due to non-CVD causes were also recorded to enable adjustment for competing events in lifetime risk analysis. Moreover, premature CVD mortality was defined as fatal CVD incidents occurring before the age of 70.

### 2.4 Evaluation of urban and rural areas as main exposure variable

The sampling frame included 78% urban and 22% rural municipalities in the Attica region (19). Based on 2001 Population and Housing Census data, the Attica region had a population of 3,761,810, which accounted for 34.3% of the total population of Greece. Of these, 2,664,776 people, or 70.8%, resided in Athens, the capital of Greece. The urban-rural gradient used in this study was based on Census 2001, as detailed in the respective meta-data. According to the Hellenic Statistical Authority, the classification of municipalities or communes as urban or rural is based on the concentrated population of their most populous settlement (i.e., the dispersed population is not taken into account). Specifically, an urban area is defined as any municipality or commune whose most populous settlement exceeds 2,000 inhabitants, while those whose most populous settlement has fewer than 2,000 residents are classified as rural.

### 2.5 Assessment of covariates

At baseline, information on socio-demographic characteristics was collected via a self-administered questionnaire (19). Specifically, the acquired data included: age, expressed in years since date of birth; biological sex, as a binary variable (males and females); marital status, categorized as a nominal variable: never married, married, and divorced or widowed; number of children; occupational status, evaluated via a 10-point ordinal rating scale ranging from unskilled to high-skilled workers; educational level, and mean annual income over the past three years, recorded as a continuous variable and categorized into four groups: low (≤€8,000), medium (€8,001–10,000), high (€10,001–20,000), and very high (>€20,000). The socio-economic status was evaluated through an index that combined the latter two variables. Specifically, three classes were constructed as follows: low class (low medium income and <9 years of education attainment, or low income and <14 years of education), high class (high very high income and ≥15 years of schooling, or very high income and 10–14 years of education), and middle class (all other combinations).

At each examination, lifestyle habits were meticulously evaluated (19–22). Specifically, participants were classified into two groups based on their smoking status: active smokers, defined as those who were smoking ≥1 cigarette daily, or non-smokers, defined as those who did not smoke during the respective examination. Moreover, participants were categorized into four trajectories based on their smoking status from 2002 to 2012: never smokers (no smoking history), continued smoking (active smokers at both examinations), started smoking (non-smokers in 2002, but active smokers in 2012), and quitted (active smokers in 2002, but non-smokers in 2012). To calculate pack-years of smoking, the number of daily cigarette packs (assuming each pack contains 20 cigarettes) was multiplied by the years of smoking. Dietary assessment was conducted using a validated 156-item semi-quantitative food frequency questionnaire (23), which recorded subjects’ consumption of food items on a daily or weekly basis over the preceding calendar year. Diet quality was evaluated employing the MedDietScore (range: 0–55), where the maximum score signifies optimal adherence to the traditional Mediterranean-type pattern (24). Additionally, participants were divided into two groups: those with low and those with high adherence to the Mediterranean dietary pattern, with the median value of 27/55 serving as the cut-off for classification. Utilizing this classification, four trajectories of Mediterranean diet adherence were constructed using data from the 2002 and 2012 examinations: sustained low (MedDietScore: <27/55 at both evaluations), low-to-high (MedDietScore: <27/55 in 2002, but ≥27/55 in 2012), high-to-low (MedDietScore: ≥27/55 in 2002, but <27/55 in 2012), and sustained high (MedDietScore: ≥27/55 at both assessments). Participation in physical activity was evaluated using the translated Short Form of the International Physical Activity Questionnaire (IPAQ-SF), which had been previously validated for the Greek population (25). An episode of physical activity was defined as any type of recreational (non-occupational) activity that lasted for a time interval of ≥10 min. Sedentary participants were those who did not report any physical activities, while active individuals were those who engaged in physical activity. The active were further classified according to the intensity of their activities, as determined by the expended calories per minute: light (<4 kcal/min), moderate (4–7 kcal/min), and high (>7 kcal/min) intensity. Accordingly, the trajectories of physical activity from 2002 to 2012 were defined as follows: always inactive (sedentary lifestyle during both examinations), became active (physically inactive in 2002, but active in 2012), became inactive (physically active in 2002, but inactive in 2012), remained active (active at both examinations).

During the baseline, biochemical measurements, different circulating biomarkers were measured according to standard protocols that have been meticulously outlined in prior publications of the ATTICA Study (19, 26). Additionally, all analyses were performed at the same laboratory designated as a reference laboratory, in accordance with WHO standards. Among the inflammatory and coagulation biomarkers measured were: high sensitivity C-reactive protein (hs-CRP), homocysteine, serum amyloid A (SAA), interleukin-6 (IL-6), serum tumor necrosis factor-alpha (TNF-α), white blood cell count, and plasma fibrinogen. Lipid profile measurements comprised: serum total cholesterol, high density lipoprotein cholesterol (HDL-C), triglycerides (TG), apolipoprotein B100 (ApoB100), ApoA1, and lipoprotein (a) [Lp(a)], with low density lipoprotein cholesterol (LDL-C) levels determined via the Friedewald formula.

The evaluated clinical entities included the presence of hypertension, hypercholesterolemia, and type 2 diabetes mellitus (19). Particularly, hypertension diagnosis was established when systolic blood pressure was ≥140 mmHg and or diastolic blood pressure was ≥90 mmHg, measured three times at the right arm using an aneroid sphygmomanometer with participants seated after a minimum of a 30 min rest period, and or when the subjects self-reported use of antihypertensive drugs. Hypercholesterolemia was confirmed in participants with total serum cholesterol levels exceeding 200 mg/dl and or those who received lipid-lowering medication. Type 2 diabetes mellitus was diagnosed with fasting blood glucose levels ≥126 mg/dl and or the prescription of oral hypoglycemic agents or insulin subcutaneous injections. Trajectories of these clinical health conditions from 2002 to 2012 were grouped as follows: presence of each condition during the 2002–2012 period; development of the condition (normal in 2002 but diagnosed in 2012); and absence of each pathological entity at both evaluations.

The anthropometric baseline measurements encompassed: body weight (in kg), height (in cm), waist circumference (in cm), and hip circumference (in cm) and were performed using standardized procedures and appropriate equipment as detailed in previous studies (19, 27). Body mass index (BMI), defined as the ratio of body mass (in kg) per squared height (in m^2^), as well as waist-to-hip (WRH) and waist-to-height (WHtR) ratios, were calculated accordingly. BMI was employed for the classification of underweight (<18.5 kg/m^2^), normal (18.5–24.9 kg/m^2^), overweight (25–29.9 kg/m^2^), and obesity (≥30 kg/m^2^) (28). Due to the very low prevalence of underweight at each examination, this category was combined with the normal BMI category. Trajectories of overweight/obesity during the 2002–2012 period included: sustained increased weight (BMI ≥ 25 kg/m^2^ at both examinations), normal BMI to overweight/obesity (BMI < 25 kg/m^2^ in 2002, but ≥25 kg/m^2^ in 2012), overweight/obesity to normal BMI (BMI ≥ 25 kg/m^2^ in 2002, but 25 kg/m^2^ in 2012), and maintenance in the normal BMI range across both examinations. Sex-specific abnormal thresholds of waist circumference were ≥88 cm for females and ≥102 cm for males [17], and abnormal values of WRH were ≥0.80 for females and ≥0.95 for males (29). The WHtR cut-off was set at ≥0.50, regardless of sex (30).

### 2.6 Statistical analysis

All statistical analyses were conducted using STATA version 18 (STATA Corp., College Station, Texas, USA). The sample size was determined to guarantee that the statistical power was sufficient to detect risk differences larger than 10%, resulting in a statistical power surpassing 80% at a *p*-value of less than 0.05 for two-sided hypotheses tested. Moreover, a *p*-value threshold of 5% was set for the two-sided tests.

Descriptive statistics are utilized to present the variables. Categorical variables are expressed as relative frequencies (*%*). Mean values, *M*, along with the corresponding standard deviations, SD, are reported for normally distributed continuous variables. Otherwise, skewed numerical variables are represented as medians, *Mdn*, with interquartile ranges, IQR, as a measure of dispersion. To test differences between two independent samples, the chi-square test was employed for categorical variables; the Student *t*-test was used for normally distributed continuous variables with equal variances; the Welch's *t*-test was applied in cases of normally distributed variables with unequal variances; and the Mann–Whitney *U* test was employed to test between-group differences for non-Gaussian continuous variables.

Crude cumulative incidence and mortality rates were determined by the ratio of the number of incident fatal or non-fatal CVD events to the total number of participants during each follow-up period. The time-to-event data was recorded on an annual basis. To evaluate the between-group differences in CVD incidence or mortality rates, the log-rank test was employed. Moreover, residual or remaining lifetime risk (LTR) estimates of experiencing a CVD episode up to the age of 85 were computed from specific index ages (*i.e.*, 40–50, 50–60, and 60–70 years), using a modified Kaplan-Meier analysis that accounts for competing risks from non-CVD deaths, as endorsed by other investigators (31, 32). Additionally, the DALYs attributed to CVD, reflecting the CVD-specific disease burden, were calculated by summing the years of life lost due to premature CVD mortality and the years of healthy life lost due to CVD-related disability.

An interaction term between biological sex and the variable indicating urban or rural residence was included in an age-adjusted Cox proportional hazard model, using the 20-year CVD incidence as the outcome, to explore the potential moderating effect of sex. Subsequently, nested Cox proportional hazard models were employed to examine the link between urban-rural areas and the 20-year CVD incidence after integrating blocks of other CVD predisposing risk factors hierarchically based on their descending effect size. The variable selection among others with similar connotations was based on selecting the model with the optimal fit, determined by the lowest values of the Akaike Information Criterion (AIC) and Bayes Information Criterion (BIC). Moreover, the Efron approximation was also incorporated to handle ties in person-years resulting from the skewed nature of the failure-time data. The statistical significance of the omnibus test was utilized to evaluate whether each block of predictors included in the model collectively enhanced the model fit compared to a null model. Moreover, the statistical significance of the likelihood ratio test was indicative of an improvement in the fit following the addition of each block of predictors.

Generalized structural equation modeling (GSEM) was utilized to explore the proposed conceptual framework linking the interplay between urban-rural areas, potential mediators, and the 20-year CVD cumulative incidence (Figure 1). Binary exogenous variables (*i.e.*, 20-year CVD incidence, clinical diagnoses, smoking status, engagement in physical activity, and adherence to the Mediterranean diet) were modeled employing Bernoulli distribution with logit link function. The selection of manifest variable combinations in the models as well as family and link functions for variables was based on achieving the lowest values of AIC and BIC while ensuring the interpretability of the models. Maximum likelihood estimation was employed to estimate coefficients and 95% confidence intervals.

## 3 Results

### 3.1 Crude analysis for 20-year CVD incidence

Among the 1,988 individuals with complete CVD data up to the 20-year of follow-up (2002–2022), a total of 718 fatal or non-fatal CVD cases were observed. The incidence rate among males was 400 per 1,000 participants, significantly higher than the 320 per 1,000 recorded in females (*p* for sex difference: <0.001). The median age at CVD was 67.0 (19.0) years for males and 70.0 (16.5) years for females (*p* for sex difference: 0.054), with a median survival time of 10 (5) years for both sexes.

### 3.2 Urban-rural gradients on CVD incidence

An association between the living environment and CVD incidence was observed. In particular, during the first decade of the study (2002–2012), new CVD cases accounted for 168 events per 1,000 participants living in urban areas and 118 per 1,000 individuals residing in rural municipalities (*p* for urban-rural difference: 0.006) (Table 1). Notably, this discrepancy was significant only among males. Regarding the entire 20-year follow-up period (2002–2022), CVD incidence increased to 387 events per 1,000 urban living participants and to 280 per 1,000 rural residents (*p* for urban-rural difference: <0.001). Both males and females living in residences in urban settings experienced higher CVD incidence rates compared to their rural counterparts.

**Table 1:** Sex- and age-specific 10- and 20-year CVD incidences, 20-year mortality, and premature CVD deaths in urban and rural samples from the ATTICA study (*n* *=* 1,988).

Epidemiological indices	Rural areas				Urban areas				*p*-value for urban-rural differences		
	Total	Males (*n* *=* 227)	Females (*n* *=* 219)	*p*-value for sex differences	Total	Males (*n* *=* 760)	Females (*n* *=* 780)	*p*-value for sex differences	Total	Males	Females
Median age at CVD, years	67 (15)	67 (15)	66 (17)	0.529	67 (20)	66 (20)	71 (17)	0.089	0.329	0.511	0.435
10-year CVD incidence, *%*	11.8	14.1	9.5	0.132	16.8	21.5	12.3	<0.001	0.006	0.007	0.237
≤35 years old	2.1	3.0	1.4		4.9	8.8	1.9				
35–45 years old	5.1	7.4	2.9		6.1	7.9	4.3				
45–55 years old	16.5	19.0	13.3		17.0	21.9	11.2				
55–65 years old	35.7	32.0	41.2		31.5	37.8	25.5				
>65 years old	42.3	31.3	46.8		55.2	60.0	63.6				
20-year CVD incidence, *%*	28.0	31.3	24.7	0.124	38.7	43.1	34.3	<0.001	<0.001	0.001	0.006
≤35 years old	2.8	3.0	2.7		6.3	9.4	3.9				
35–45 years old	5.8	8.7	2.9		7.3	10.2	4.3				
45–55 years old	49.0	50.0	47.8		53.5	59.2	46.6				
55–65 years old	97.5	100.0	94.1		96.2	100.0	93.2				
>65 years old	100.0	100.0	100.0		100.0	100.0	100.0				
20-year CVD mortality, *%*	3.7	6.0	1.2	0.005	4.8	7.7	1.9	<0.001	0.082	0.348	0.481
Premature CVD mortality, *%*	2.1	4.1	0	0.002	2.4	3.9	1.0	0.001	0.683	0.814	0.126

*p*-values were derived using the log-rank test for incidence rates and the Mann–Whitney *U* test for the median ages, respectively.

Stratified sex-centric analyses for rural and urban areas revealed significant between-sex differences only among individuals living in urban regions, with males exhibiting greater incidence rates than females, by 9.0% and 8.8% for the 10- and 20-year follow-ups, respectively (*p*s for sex differences: <0.001) (Table 1). In both urban and rural municipalities, age-standardized male-to-female 10-year incidence rate ratios revealed that males exhibited more than double incidence rates of females up to the age of 55 years. However, this difference was attenuated to approximately 1.5 in the 55–65 age group, and then it was even reversed, with greater CVD incidence rates recorded in females >65 years old than in men. Regarding the crude 20-year cumulative incidence, sex-specific disparities were apparent up to the age bracket of 35–45 years old, with higher incidence rates demonstrated in men. Nevertheless, these discrepancies were mitigated, resulting in almost equal rates for both sexes after this age. Overall, during the 20-year study period, more fatal CVD events, whether premature or not, were recorded in males than in females, in both urban and rural settings.

Although the residual or remaining LTR of developing a fatal or non-fatal CVD event was comparable for urban and rural inhabitants across all index ages, significant between-sex urban-rural differences emerged (Table 2). Specifically, urban living females exhibited elevated LTR at the index age of 40 in comparison to rural living females. Conversely, rural males exhibited a greater LTR at the index age of 60 for developing CVD through age 85 years compared to males residing in urban regions. Sex-based analyses for participants living in urban and rural settings suggested that at index ages of 40 and 50, males had a greater chance of developing an incident CVD event than females. Among rural inhabitants, this sex-specific disparity diminished as the participants aged without experiencing any CVD episode, indicating comparable residual lifetime CVD risk estimates for both sexes measured from the index age of 60. However, in urban agglomerations, the estimated LTR at the index age of 60 was greater for females than in males. As for the CVD burden, DALYs attributed to CVD were similar among those who resided in rural and urban settings, irrespective of sex.

**Table 2:** Sex- and age-specific lifetime risk estimates (probabilities of developing a CVD event throughout life course) and DALYs in participants from the ATTICA study residing in urban and rural regions (*n* *=* 1,988).

Epidemiological indices	Rural areas				Urban areas				*p*-value for urban-rural differences		
	Total	Males (*n* *=* 227)	Females (*n* *=* 219)	*p*-value for sex differences	Total	Males (*n* *=* 760)	Females (*n* *=* 782)	*p*-value for sex differences	Total	Males	Females
Lifetime risk, *%* (95% CI)											
40–50	69.9 (68.1, 71.7)	76.6 (74.8, 78.4)	62.2 (59.7, 64.6)	<0.001	70.8 (69.8, 71.7)	76.0 (74.9, 77.0)	65.3 (63.9, 66.7)	<0.001	0.377	0.555	0.030
50–60	64.9 (62.7, 67.2)	67.7 (65.1, 70.4)	60.9 (57.2, 64.6)	0.002	64.9 (63.9, 65.9)	67.0 (65.7, 68.3)	62.7 (61.2, 64.1)	<0.001	0.947	0.606	0.358
60–70	67.2 (63.3, 71.1)	70.6 (64.6, 76.6)	63.5 (58.5, 68.4)	0.066	65.2 (63.8, 66.7)	63.6 (61.6, 65.6)	66.6 (64.6, 68.6)	0.039	0.307	0.009	0.275
DALYs (95% CI)	12.9 (10.9, 14.9)	11.5 (8.9, 14.2)	14.8 (11.7, 17.9)	0.113	12.2 (11.4, 13.1)	12.6 (11.3, 13.9)	11.9 (10.7, 13.1)	0.431	0.540	0.502	0.061

*p*-values were derived using the Student *t*-test and the Welch's *t*-test for normally distributed variables with equal and unequal variances, respectively.

Lifetime risk denotes the percentage of individuals expected to encounter a fatal or non-fatal event from each age-index until the end of the 20-year follow-up, assuming the last cohort participants survives up to 85 years of age. DALYs: disability-adjusted life years, 95% CI: 95% confidence interval.

### 3.3 Sex-based urban-rural differences in determinants of the 20-year CVD incidence

The median age at CVD of participants living in urban municipalities was substantially higher than that of rural inhabitants for both sexes (Table 3). When examining urban-rural differences in the 10-year trajectories of major cardiometabolic risk factors between 2002 and 2012, only a discrepancy emerged in the trajectories of hypertension among females. Specifically, the prevalence of hypertension at both assessments was greater among females residing in urban municipalities, whereas the percentages of females who remained normotensive or were newly diagnosed with elevated blood pressure were higher in rural females. Moreover, males dwelling in urban areas exhibited elevated plasma fibrinogen and homocysteine levels in comparison to males living in rural areas.

**Table 3:** Urban-rural differences regarding clinical, anthropometric, and laboratory circulating CVD predisposing factors among participants of the ATTICA study (*n* *=* 1,988).

Variables	Rural areas				Urban areas				*p*-value for urban-rural differences	
	Total	Males (*n* = 227)	Females (*n* = 219)	*p*-value for sex differences	Total	Males (*n* = 760)	Females (*n* = 782)	*p*-value for sex differences	Males	Females
Age at baseline, years	42 (16)	43 (17)	41 (16)	0.494	46 (19)	46 (18)	45 (20)	0.146	<0.001	<0.001
Hypertension (2002–2012), *%*				<0.001				<0.001	0.480	0.023
Hypertension during 2002–2012	44.1	56.4	29.3		46.6	53.1	40.0			
Developed hypertension =	10.9	7.7	14.7		10.6	10.2	10.9			
Normal blood pressure =	45.0	35.9	56.0		42.8	36.7	49.1			
Hypercholesterolemia (2002–2012), *%*				0.010				0.010	0.226	0.279
Hypercholesterolemia during 2002–2012	55.8	62.5	48.4		56.5	58.6	54.3			
Developed hypercholesterolemia	8.5	7.1	10.1		9.5	10.7	8.3			
Normal serum total cholesterol	35.7	30.4	41.5		34.0	30.7	37.4			
Diabetes mellitus (2002–2012), *%*				0.064				0.474	0.984	0.097
Diabetes mellitus during 2002–2012	10.3	13.6	6.9		12.2	13.2	11.0			
Developed diabetes mellitus	10.0	11.4	8.6		11.8	11.7	12.0			
Normal fasting blood glucose levels	79.7	75.0	84.5		76.0	75.1	77.0			
Overweight obesity (2002–2012), *%*				<0.001				<0.001	0.388	0.206
Sustained increased weight	31.9	40.7	23.0		35.9	47.4	25.2			
Normal BMI to overweight obesity	28.0	18.1	38.0		24.9	16.2	33.0			
Overweight obesity to normal BMI	21.2	28.2	14.1		22.3	25.4	19.4			
Remained in normal BMI range	18.9	13.0	24.9		16.9	11.0	22.4			
BMI (2012), kg/m^2^	26.3 ± 4.5	26.2 ± 4.5	26.4 ± 4.4	0.657	26.7 ± 4.4	26.9 ± 4.5	26.5 ± 4.3	0.051	0.050	0.898
Waist circumference (2002), cm	90.5 ± 16.3	97.7 ± 15.2	82.5 ± 13.6	<0.001	90.1 ± 14.9	97.7 ± 12.4	82.9 ± 13.4	<0.001	0.997	0.727
WHR (2002)	0.87 ± 0.12	0.93 ± 0.12	0.80 ± 0.07	<0.001	0.86 ± 0.11	0.93 ± 0.09	0.80 ± 0.09	<0.001	0.912	0.801
WHtR (2002)	0.53 ± 0.09	0.56 ± 0.09	0.51 ± 0.09	<0.001	0.53 ± 0.08	0.56 ± 0.07	0.51 ± 0.08	<0.001	0.633	0.772
Fibrinogen (2002), mg/dl	298 (83)	291 (82)	305 (84)	0.001	304 (88)	296 (88)	311 (90)	<0.001	0.025	0.345
Homocysteine (2002), μmol/L	10.6 (4.3)	11.4 (4.6)	9.9 (3.4)	<0.001	10.9 (4.3)	11.9 (4.3)	9.7 (3.9)	<0.001	0.005	0.738
hs-CRP (2002), mg/L	1.04 (2.10)	1.15 (1.94)	0.94 (2.23)	0.034	1.04 (1.85)	1.11 (1.69)	0.95 (1.95)	0.027	0.246	0.955

Continuous variables are reported as means (*M*) with standard deviations (SD), denoted as *M* ± SD, when normality assumptions were satisfied. Otherwise, medians (*Mdn*) with interquartile ranges (IQR), as Mdn (IQR), are utilized to report numerical variables. Categorical variables are expressed as relative frequencies (*%*).

*p*-values were calculated employing the Student *t*-test for normally distributed continuous variables with equal variances, the Welch's *t*-test for normally distributed variables with unequal variances, the Mann-Whitney U test for skewed numerical variables, and the chi-square test for categorical variables.

BMI, body mass index; hs-CRP, high-sensitivity C-reactive protein; WHtR, waist-to-height ratio; WHR, waist-to-hip ratio.

Additionally, urban-rural differences were found only in females regarding the trajectories to the Mediterranean diet adherence, with a greater proportion of females in urban areas sustaining low adherence during the 20-year time window, while a greater percentage of females in rural regions sustained high adherence to the Mediterranean-type dietary pattern (Table 4). At the 10-year follow-up, the percentages of participants with high adherence to the Mediterranean diet and the median values of MedDietScore were increased among rural residents than their urban counterparts for both sexes. Likewise, the 10-year trajectories of physical activity exhibited urban-rural discrepancies only among men, with a greater percentage of rural males remaining physically active compared to their urban counterparts. Furthermore, in 2012, 57.1% of males in rural municipalities were physically active, whereas the respective percentage of physically active males in urban agglomerations was 19.4%.

**Table 4:** Urban-rural differences in lifestyle-related CVD predisposing factors, and socio-economic status among participants of the ATTICA study (*n* *=* 1,988).

Variables	Rural areas				Urban areas				*p*-value for urban-rural differences	
	Total	Males (*n* *=* 227)	Females (*n* *=* 219)	*p*-value for sex differences	Total	Males (*n* *=* 760)	Females (*n* *=* 782)	*p*-value for sex differences	Total	Men (*n* *=* 227)
Smoking habits (2002–2012), *%*				0.310				0.012	0.222	0.874
Never smokers	42.0	41.9	42.2		37.5	35.6	39.5			
Continued smoking	19.5	20.8	18.1		21.9	24.7	19.1			
Started smoking	19.1	16.2	22.1		20.7	19.1	22.3			
Quitted smoking	19.4	21.1	17.6		19.9	20.6	19.1			
Active smokers (2012), *%*	38.5	37.0	40.2	0.459	42.5	43.7	41.4	0.309	0.050	0.734
Mediterranean diet (2002–2012), *%*				<0.001				<0.001	0.163	0.004
Sustained low	21.1	32.6	8.9		27.7	39.4	16.1			
Low-to-high	9.7	15.6	3.3		9.5	15.6	3.5			
High-to-low	46.8	44.8	49.1		45.2	39.6	50.7			
Sustained high	22.4	7.0	38.7		17.6	5.4	29.7			
High adherence to Mediterranean diet (2012), *%*	34.0	12.2	57.2	<0.001	25.9	8.1	43.4	<0.001	0.032	<0.001
MedDietScore (2012), 0–55 units	26.1 (2.9)	25.2 (2.1)	27.3 (2.5)	<0.001	25.6 (3.0)	24.8 (2.0)	26.6 (2.8)	<0.001	0.001	0.001
Physical activity (2002–2012), v*%*				0.270				0.925	0.010	0.566
Always inactive	40.0	42.9	33.4		56.8	55.9	57.8			
Became active	0	0	0		8.5	7.5	9.6			
Became inactive	10.0	0	33.3		23.3	24.7	21.7			
Remained active	50.0	57.1	33.3		11.4	11.8	10.9			
Physically active (2012), *%*	50.0	57.1	33.4	0.490	19.9	19.5	20.5	0.852	0.020	0.591
Socioeconomic status, *%*				0.690				0.004	0.896	0.099
Low class	17.1	15.7	19.0		14.7	15.1	14.3			
Middle class	49.1	49.8	48.2		52.7	48.7	56.9			
High class	33.8	34.5	32.8		32.6	36.2	28.8			

Continuous normally distributed variables are expressed as means (*M*) with standard deviations (SD), as *M* ± SD; otherwise, medians (*Mdn*) with interquartile ranges (IQR), as Mdn (IQR). Categorical variables are reported as relative frequencies (*%*). *p*-values were calculated employing the Student *t*-test for normally distributed continuous variables with equal variances, the Welch's *t*-test for normally distributed variables with unequal variances, the Mann–Whitney *U* test for skewed numerical variables, and the chi-square test for categorical variables.

Independent assessments of urban and rural settings revealed significant between-sex differences in the trajectories of hypertension and hypercholesterolemia (Table 3). Particularly, in both settings, greater proportions of males were diagnosed with these conditions at both examinations, while larger percentages of females maintained normal blood pressure and total cholesterol levels during this time window. Moreover, in both urban and rural municipalities, although a higher percentage of males sustained an increased BMI during the first decade of the study and had greater baseline anthropometric indices compared to females, a greater share of males returned to normal BMI ranges and a lower proportion of males transitioned from a normal BMI to overweight/obese status in comparison to females. Similarly, in urban and rural municipalities, despite higher percentages of males sustaining low adherence to the Mediterranean-type diet and lower percentages of males with high adherence compared to females from 2002 to 2012, the percentage of males who increased their adherence to the Mediterranean diet was almost five times larger than that of females (Table 4). However, in 2012, a greater proportion of females had a MedDietScore above the cut-off of 27/55 and females achieve higher median score in the MedDietScore compared to men, irrespective of whether they lived in urban or rural areas. Additionally, only in urban regions, the 10-year trajectories of smoking and the distribution among socio-economic classes unraveled significant sex-based discrepancies.

### 3.4 Multivariate analysis of the role of built environment on CVD risk

The potential moderating effect of sex on the interplay between urban-rural disparities and 20-year CVD incidence was investigated by integrating an interaction term between sex and the variable of urban-rural areas in an age-adjusted Cox proportional hazard model. Although the interaction term was found to be statistically significant (*p* for interaction: <0.001), urban-rural disparities were observed only for males [hazard ratio (HR): 1.79, 95% CI: 1.21–2.62; *p* for urban-rural difference: 0.003], whereas the respective term did not reach statistical significance for females (HR: 0.86, 95% CI: 0.58–1.29; *p* for urban-rural difference: 0.487).

Additionally, to mitigate potential biases from residual confounding due to the absence of adjustments for other CVD predisposing factors, sex-specific nested Cox proportional hazard models were employed to further explore these relationships (Table 5). Among men, the variable of urban-rural disparities lost its significance when the lifestyle-related risk factors were entered into the model, suggesting that lifestyle-related risk factors may mediate the relationship between urban agglomerations and 20-year CVD incidence. Notably, adherence to the Mediterranean diet emerged as the sole substantial cardio-protective lifestyle factor.

**Table 5:** Results from nested Cox proportional hazard models investigating urban-rural disparities, along with the effect of age, clinical, and modifiable predisposing factors on the long-term (20-year) CVD incidence in participants of the ATTICA study (*n* = 1,988).

	Model 1	Model 2	Model 3	Model 4
Females				
Urban-rural settings, ref: rural areas	1.88 (1.17, 3.00)**	1.36 (0.65, 2.87)	1.24 (0.55, 2.79)	1.18 (0.52, 2.69)
Age at baseline, per 1 year		1.31 (1.24, 1.37)**	1.28 (1.21, 1.35)***	1.28 (1.21, 1.35)***
Hypertension (2012), ref.: no			2.11 (1.03, 4.32)*	2.31 (1.10, 4.84)*
Hypercholesterolemia (2012), ref.: no			4.51 (2.14, 9.51)***	4.39 (2.08, 9.29)***
Diabetes mellitus (2012), ref: no			8.02 (2.88, 22.4)***	8.42 (2.98, 23.8)***
WHtR (2002), per 1 unit			0.12 (0.001, 28.3)	0.07 (0.001, 17.8)
Active smoking (2012), ref.: no				1.87 (0.92, 3.80)
MedDietScore (2012), per 1/55 unit				0.99 (0.92, 1.07)
Physical activity (2002), ref.: sedentary				1.04 (0.92, 1.07)
*p*-value (omnibus test)	0.011	<0.001	<0.001	<0.001
*p*-value (likelihood ratio test)	0.007	<0.001	<0.001	0.328
Males				
Urban-rural settings, ref: rural areas	2.33 (1.48, 3.69)***	2.09 (1.05, 4.16)*	2.23 (1.06, 4.68)*	1.96 (0.91, 4.22)
Age at baseline, per 1 year		1.33 (1.26, 1.39)***	1.31 (1.24, 1.38)***	1.32 (1.25, 1.40)***
Hypertension (2012), ref.: no			2.18 (1.18, 4.02)*	2.26 (1.21, 4.20)*
Hypercholesterolemia (2012), ref.: no			2.13 (1.15, 3.93)*	2.80 (1.11, 3.90)*
Diabetes mellitus (2012), ref: no			2.90 (1.40, 6.02)**	3.07 (1.45, 6.51)**
WHtR (2002), per 1 unit			0.91 (0.01, 55.9)	0.36 (0.01, 21.6)
Active smoking (2012), ref.: no				1.66 (0.90, 3.03)
MedDietScore (2012), per 1/55 unit				0.89 (0.82, 0.97)**
Physical activity (2002), ref.: sedentary				1.01 (0.55, 1.87)
*p*-value (omnibus test)	0.001	<0.001	<0.001	<0.001
*p*-value (likelihood ratio test)	0.001	<0.001	<0.001	0.019

Results are expressed as hazards ratios (HR) and 95% confidence intervals (95% CI). The combination of risk factors that resulted in the optimal model fit, determined by the lowest values of the Akaike Information Criterion and Bayesian Information Criterion, was selected.

Model 1: Urbanization.

Model 2: Model 1 + Age at enrollment + Socioeconomic status.

Model 3: Model 2 + Hypertension (2012) + Hypercholesterolemia (2012) + Diabetes mellitus (2012) + WHtR (2002).

Model 4: Model 3 + Active smoking (2012) + MedDietScore (2012) + Physical activity (2002).

*** *p-*value < 0.001, **p-value < 0.01, *p-value < 0.05. WHtR, waist-to-height ratio.

To further elucidate the intricate pathways between urban-rural disparities, lifestyle-related risk factors, and the 20-year CVD incidence, a mediation analysis was conducted. The results of the GSEM are depicted in Figure 1; Table 6. Living in an urban area is associated with a 90% [odds ratio (OR): 1.90, 95% CI: 1.05–3.39] higher risk of developing a fatal or non-fatal incident CVD event during a 20-year period, compared to living in a rural region. Moreover, participants residing in urban areas exhibit a 32% (OR: 0.68, 95% CI: 0.55–0.83) lower adherence to the Mediterranean diet than their counterparts dwelling in rural settings. Subsequently, subjects with higher adherence to the Mediterranean diet presented a lower risk of being diagnosed with major cardiometabolic CVD risk factors, thus suggesting that greater adherence to the Mediterranean diet provides an indirect cardio-protective effect on 20-year CVD incidence among urban participants.

**Table 6:** Results from the generalized structural equation modeling investigating the interplay between urban-rural disparities, major clinical risk factors, lifestyle-related predisposing factors, and the 20-year CVD incidence in participants of the ATTICA study (*n* = 1,988).

Outcome variables	Manifest variables	Path coefficients (95% CI)
20-year CVD incidence, ref.: no		
	Age at baseline, per 1 year	0.16 (0.13, 0.20)***
	Hypertension (2012), ref.: no	0.75 (0.26, 1,23)**
	Hypercholesterolemia (2012), ref.: no	0.59 (0.10, 1.08)*
	Diabetes mellitus (2012), ref: no	1.38 (0.81, 1.95)***
	Overweight obesity (2022), ref: no	−0.06 (−0.57, 0.45)
	Urban-rural settings, ref: rural areas	0.64 (0.05, 1,22)**
Hypertension (2012), ref.: no		
	Age at baseline, per 1 year	0.06 (0.05, 0.07)***
	Active smoking (2012), ref.: no	−0.01 (−0.24, 0.22)
	Adherence to the Mediterranean diet (2012), ref.: low	−1.10 (−1.38, −0.81) ***
	Physical activity (2002), ref.: sedentary	−0.21 (−0.43, 0.02)
Hypercholesterolemia (2012), ref.: no		
	Age at baseline, per 1 year	0.06 (0.05, 0.07)***
	Active smoking (2012), ref.: no	0.21 (−0.01, 0.44)
	Adherence to the Mediterranean diet (2012), ref.: low	−0.60 (−0.86, −0.35)***
	Physical activity (2002), ref.: sedentary	−0.28 (−0.50, −0.06)*
Diabetes mellitus (2012), ref: no		
	Age at baseline, per 1 year	0.07 (0.06, 0.08)***
	Active smoking (2012), ref.: no	0.19 (−0.07, 0.46)
	Adherence to the Mediterranean diet (2012), ref.: low	−0.90 (−1.35, −0.45)***
	Physical activity (2002), ref.: sedentary	−0.53 (−0.81, −0.25)***
Overweight obesity (2022), ref: no		
	Age at baseline, per 1 year	−0.01 (−0.02, 0.01)
	Active smoking (2012), ref.: no	−0.23 (−0.49, 0.02)
	Adherence to the Mediterranean diet (2012), ref.: low	−2.55 (−2.83, −2.27)***
	Physical activity (2002), ref.: sedentary	−0.46 (−0.72, −0.21)***
Adherence to the Mediterranean diet (2012), ref.: low		
	Urban-rural settings, ref: rural areas	−0.39 (−0.59, −0.19)***
Active smoking (2012), ref.: no		
	Urban-rural settings, ref: rural areas	0.17 (−0.03, 0.37)
Physical activity (2002), ref.: sedentary		
	Urban-rural settings, ref: rural areas	0.04 (−0.14, 0.21)

*** *p-*value < 0.001, **p-value < 0.01, *p-value < 0.05.

## 4 Discussion

Prior research has already explored the role of the built environment in CVD risk. This study sought to further evaluate sex-related research gaps by exploring urban-rural disparities in CVD epidemiology and sex-related risk factors in a nationally representative sample of Greek individuals over the course of a 20-year observation period. It was revealed that urban living environment was associated with higher middle- and long-term incidences of CVD as compared to rural environment. Sex-related discrepancies were observed, highlighting the crucial role of sex in better understanding CVD epidemiology. Moreover, multivariate analyses revealed that the impact of the urban built environment on CVD risk is mediated by lifestyle-related risk factors. The built environment is closely associated with behaviors and exposures that may influence cardiovascular health. By designing and maintaining healthy environments, communities have the potential to support reduced CVD incidence and promote overall well-being. Achieving this goal requires a multidisciplinary approach, involving urban planners, public health professionals, policymakers, and community members.

### 4.1 Main findings

The ATTICA Study is the first prospective cohort study in the Mediterranean peninsula, and one of the few worldwide systematically collecting CVD-related data at multiple time points over a long period (2002–2022). In the present analysis, it was observed that the 10-year CVD incidence was approximately 40% higher in people living in urban municipalities as compared to rural (*i.e.*, 16.8% vs. 11.8%) and this trend remained similar, *i.e.*, 38%, at 20-year follow-up (38.7% in urban municipalities vs. 28.0% in rural). The previous findings were irrespective of the age of the participants but seem to be partially explained by the sex of the participants as well as lifestyle-related behaviors. Notably, only males living in urban regions exhibited a higher 10-year CVD risk than rural men, whereas no differences were observed among females. As participants grew older over the 20-year follow-up period, the number of CVD events increased similarly for both sexes, with individuals living in urban areas continuing to demonstrate higher CVD incidence rates than their rural counterparts. In addition, age-standardized male-to-female analyses revealed that the higher male-specific CVD incidence rates were diminished at the age of 45, with both sexes presenting almost equal incidence rates beyond that age. This pattern was consistent in both urban and rural areas. Similarly, LTR estimates at index ages of 40 and 50 suggested that urban-living males had a greater chance of developing an CVD event than females during their remaining lifespan; however, this sex-specific disparity was attenuated at the index age of 60. Recently, the ATTICA Study investigators employed Geographic Information Systems (GIS) and spatial statistical methods to investigate geographical disparities in 10- and 20-year CVD incidence rates across the Attica region (18). The analysis revealed significant spatial heterogeneity in CVD incidence within the studied region, with the clustered central-eastern parts of Attica, the most urbanized areas, exhibiting notably higher CVD incidence rates.

### 4.2 Understanding the role of built environment on CVD incidence

In a systematic review and meta-analysis that explored the urban-rural gradient in relation to CVD outcomes, Angkurawaranon et al. (33) found that urban inhabitants presented a 2.48-times, i.e., 148% (95% CI: 1.20–5.11) higher odds to being diagnosed with coronary heart disease compared to their rural counterparts (34). However, this meta-analysis was based only on seven cross-sectional studies conducted in countries of Southeast Asia, which restrict any causal inference, are subject to potential biases such as reverse causality, and limit direct comparability with the present study. The latest data from two Mediterranean countries indicated that between 2003 and 2019, the decline in ischemic heart disease mortality rates was 4.0% and 3.6% in urban and non-urban inhabitants in Italy (35). In Spain, these decreases were approximately 4.4% in urban areas and 3.7% in non-urban regions.

Although the exact mechanisms by which urban exposome impacts CVD risk are not yet fully understood, two main interrelated pathways have been suggested in the existing scientific literature (10). The most widely accepted mechanism linking CVD risk mitigation to greenspaces is mediated via physical activity, since close proximity to parks and physical activity facilities encourages an active lifestyle (10). Moreover, residential proximity to green areas has been associated with reduced psychological distress and better mental health (10, 36, 37), as well as enhanced social connections and cohesion, thereby fostering well-being (10). In a recent systematic review, it was reported that the urban built environment's prominent attributes, such as greenness and neighborhood-level walkability, have a protective association against CVD risk factors, including elevated blood pressure and arterial stiffness, as well as fatal or non-fatal cardiac events (10). Over the years of research, count, distance, and coverage of green areas; density of vegetation and wooded environment; and several vegetation indices have been utilized as estimates of greenness. The investigators of another systematic review and meta-analysis used the normalized difference vegetation index as an exposure measure and found that urban greenspaces provide cardio-protective effects against CVD death rates (HR: 0.94, 95% CI: 0.91–0.97), ischemic heart disease-specific mortality (HR: 0.96, 95% CI: 0.93–0.99), and cerebrovascular disease mortality (HR: 0.96, 95% CI: 0.94, 0.97) (38). Additional characteristics of the built environment that have also been examined include: the proximity to greenspaces, the number and distance of public parks, the density of residents or sports facilities, multi-scalar residential density, the distance to major roads, compactness as a function of residential density, mixed land use, street connectivity, and centeredness, as well as impervious surfaces and man-made land cover type. The prevailing mechanism between CVD health and greenspaces is an increase in physical activity levels (10), thereby enhancing cardio-respiratory fitness and reducing cardiometabolic predisposing factors (39), and thus preventing CVD events (10, 40). Notably, although females tend to engage less in physical activities than males (41), this gender gap tends to be less pronounced when females live in urban settings with high walkability (42). However, females have a lower willingness to walk in empty streets that are perceived as unsafe in comparison to men, thus lowering their physical activity levels (43). Furthermore, the investigators of another study found that in areas with greenspaces, a larger share of females participate in lower-intensity physical activity compared to men, while males exceed females in all other intensity levels (44). Moreover, residing near green areas alleviates psychological stress and improves mental health (10, 36, 37) and social interactions (10). Psychological stress, anxiety, and depression increase the risk of developing CVD, mainly in females (2).

Increasing evidence indicates that rapid urbanization, rising affluence, and other factors influence the neighborhood-built environment, thereby shaping the food environment, which is characterized by the accessibility and availability of “food resources” (9, 45). This, in turn, influences lifestyle behavioral factors, such as dietary choices and habits, and indirectly affects CVD-related outcomes (13). Specifically, food deserts and food swamps represent two sides of the same coin, resulting in limited access to an abundance of healthful foods (13). Additionally, previous studies have demonstrated that living in areas with a higher density or closer proximity to supermarkets or other retailers is associated with healthier dietary choices (45), leading to lower prevalences of cardiometabolic disorders like metabolic syndrome (39), due to better access to healthy, whole foods. Indeed, a previous analysis of the ATTICA Study revealed that the protective potential of the food environment for metabolic syndrome was about 1.5 times more pronounced in females compared to males (39). Conversely, the investigators of a recent systematic review demonstrated that the opposite is true for regions with fast-food restaurants, which led to an increase in an obesogenic environment, increasing the CVD burden (46).

Another pathway through which the urban built environment exerts its detrimental effects on CVD outcomes is exposure to various environmental stressors. A recent estimate of the GBD 2021 study reported that ambient fine particulate matter (PM) with 2.5 μm or less in diameter (PM_2.5_) was the leading environmental risk factor for premature CVD mortality, accounting for 4.75 million (95% CI: 3.76–5.58) CVD deaths in 2021 (47). Subsequently, 1.55 million (95% CI: 0.14–3.17) CVD deaths were attributed to lead exposure, followed by non-optimal ambient temperatures, which were responsible for 1.17 million (95% CI: 1.07–1.29) CVD deaths in 2021. These environmental stressors entail: airborne pollutants, such as PM_2.5_, including ultra-fine fractions with <0.1 μm diameter (PM_0.1_), and coarse particles with diameters between 2.5 μm and 10 μm (PM_10–2.5_), as well as gaseous gases, *e.g.*, carbon monoxide (CO), nitrogen oxides (NO_x_), sulfur dioxide (SO_2_), ozone (O_3_), and volatile organic compounds (VOC) (9, 48); residential noise pollution (9); non-optimal ambient temperature (9, 48) and its fluctuations; relative humidity (48); soil and water pollution, including exposure to heavy metals and toxic organic chemicals (49); as well as light pollution (9). A recent umbrella review and meta-meta-analysis found strong evidence linking exposure to ambient air pollution, particularly PM_2.5_ and residential noise, with an increase in CVD risk and highly suggestive evidence between increased ambient temperature and CVD risk (9). Although these stressors also affect rural inhabitants, urban climate conditions exacerbate their severity, inducing an urban-rural gradient. Additionally, although outdoor pollution affects both males and females, indoor pollution arising from cooking fuels disproportionately affects females, highlighting a potential under-recognized gender-specific disparity (2). Several pathogenetic pathways have been proposed to support these linkages; most of them have common denominators that are not limited to: interaction between the peripheral and central nervous system; signal transduction along the neuronal-cardiovascular axis and activation of the sympathetic cardiac autonomic nervous system; gut microbiota alternations; circadian rhythm disruptions; and peripheral-induced inflammation and oxidative stress that reach a systematic level (9, 48). These pathophysiological processes lead to pathological cascades that increase the risk of developing CVD through various mechanisms, such as endothelial dysfunction, dyslipidaemia, insulin resistance, hypercoagulability, and subclinical atherosclerosis (9, 48). Beyond shaping their own micro-climate (16), urban environments are inherently interconnected with broader regional climates through atmospheric molecules and phenomena (48); thereby, there is a bidirectional influence where urban climates not only impact but are also influenced by broader climate change phenomena (50).

In the present study, urban-rural differences were more pronounced in lifestyle-related risk factors, particularly adherence to the Mediterranean diet and engagement in physical activity, with urban individuals exhibiting lower adherence to healthy behaviors. Multivariate mapping via GSEM analysis showcased that the complex relationship between living in urban areas and 20-year CVD incidence was mediated by lower adherence to a healthy dietary pattern, such as the Mediterranean diet, highlighting the substantial impact of food environment in shaping long-term CVD outcomes by influencing lifestyle behaviors (39, 45). Specifically, urban inhabitants had poorer dietary choices, as determined by lower adherence to the traditional Mediterranean diet, thus increasing their susceptibility to major cardiometabolic risk factors, which subsequently increased the risk of experiencing a non-fatal or fatal CVD event. In addition to this path, living in an urban built environment presented a direct effect on 20-year CVD incidence, suggesting the presence of other underlying attributes of an urban built environment not included in this analysis that contribute to discrepancies observed between urban and rural populations in long-term CVD outcomes. Both air pollution and the lack of green spaces, factors that have already been mentioned for their impact on CVD incidence, are prominent environmental features of the urban areas in the Attica region. The European Environment Agency reports that over 90% of Greece's urban population is exposed to PM_10_ and O_3_ that exceed EU standards (51). Athens, the most urbanized city in Greece, ranks lowest among European capital cities for green urban areas. It is characterized by a densely built environment where buildings and infrastructure dominate the landscape, leaving minimal room for green spaces. This urban design results in several negative health impacts. The scarcity of green areas leads to reduced opportunities for Athen's residents to engage in physical activities such as walking, jogging, and outdoor sports, which are essential for maintaining cardiovascular health (52).

### 4.3 Need for action

All these findings highlight the need for the implementation of community-level programs, strategies, and policies that target primordial and primary CVD prevention under the prism of sustainable development, equity, social inclusion, and well-being, in line with the “*Healthy Cities*” initiative advocated by WHO (53). According to forecasts based on the GBD Study 2021, the age-standardized DALYs rate attributed to CVD is projected to decline to 3,215.2 (95% UI: 2,412.3–4,304.8) DALYs per 100,000 population by 2050 under a probabilistic business-as-usual (BAU) optimistic scenario (54). Following a “safer environment” scenario by 2050, which entails more aggressive actions to mitigate environmental health risks than the BAU scenario, such as achieving net zero emissions and eliminating unsafe water, sanitation, hygiene, and household air pollution by 2050, the age-standardized DALYs rate is expected to decrease to 3,103.8 (95% UI: 2,331.4–4,149.2) DALYs per 100,000 population. In the “improved childhood nutrition and vaccination” scenario, in which growth failure, vitamin deficiencies, and suboptimal breastfeeding will be eliminated and 100% vaccine coverage for key diseases will be achieved by 2050, the age-standardized DALYs rate will reach 3,214.8 (95% UI: 2,412.1–4,304.4) DALYs per 100,000 people. Nevertheless, under the “improved behavioral and metabolic risks” scenario alone, which aims to eliminate high levels of blood pressure, LDL-C, and fasting plasma glucose, as well as high BMI and smoking, as well as improve diet quality by 2050, the projected age-standardized DALYs rate is anticipated to reach 1,246.1 (95% UI: 1,019.8–1,539.0) DALYs per 100,000 population by 2050. The “combined” scenario, which integrates all the aforementioned measures, exhibits the most substantial decrease in the age-standardized DALYs rate, lowering further to 1,226.2 (95% UI: 1,005.2-1,511.3) DALYs per 100,000 people. This notable decline is primarily driven by expected improvements under the “improved behavioral and metabolic risks” scenario, resulting in a 10%–15% decrease in DALY count in countries of the Mediterranean peninsula including Greece. Conversely, the “safer environment” scenario contributes only a decrease of ≤1% in DALY counts in Mediterranean countries. These findings highlight the critical need for multidimensional strategies that prioritize lifestyle changes on future initiatives addressing primordial and primary CVD prevention, such as building greenspaces and supporting terrestrial healthy and sustainable dietary patterns, such as the Mediterranean (55).

Urban settings provide ideal venues to test community-level risk-minimizing interventions, such as “Healthy Cities,” since they are more promising and cost-effective than interventions at the individual level. Nonetheless, there is limited knowledge regarding the effect of specific attributes of the urban built environment on CVD morbidity and mortality. Furthermore, current evidence largely stems from cross-sectional studies (10), precluding any causal inference (34), while there is a paucity of research employing a prospective design that measures exposures at multiple data points and utilizes a long-term follow-up (10). Furthermore, there is insufficient evidence regarding the urban-rural gradient in European countries. The current body of research has not thoroughly examined the potential causal pathways between urban and rural environments and CVD risk.

### 4.4 Strengths and limitations

The ATTICA Study is the first prospective cohort study in the Mediterranean peninsula, collecting data at multiple time points over a 20-year period. At each examination, the sample was large and nationally representative of the Greek population, although urban areas may have been overrepresented and rural areas underrepresented. However, some weaknesses may exist, including measurement errors and data reliability issues due to the prospective design of the study, as well as recall and social-desirability biases inherent in self-reported assessments. Additionally, the sampling method, based on the age-sex distribution of the Greek population, resulted in an overrepresentation of younger adults, thereby obscuring the number of observed CVD events.

## 5 Conclusion

The results of this study provide insights into CVD epidemiology related to urban-rural environment and sex-based disparities. Therefore, there is an emerging need for community-level, public health multicomponent interventions prioritizing lifestyle factors, while addressing socio-cultural norms that may hinder prevention. By tailoring strategies to meet the needs of different communities and demographics, public health initiatives can be more effective in reducing the incidence and impact of CVD.

## Data Availability

The raw data supporting the conclusions of this article will be made available by the authors, without undue reservation.
